# Preparing the Mouth for Full Sets of Artificial Teeth

**Published:** 1853-10

**Authors:** 


*Preparing the Mouth for Full Sets of Artificial Teeth.*—Dr. A. T. Willard, of Chelsea, Mass., in an article on this subject, given in the July No. of the News-Letter, Vol. 6, recommends the following practice:

"After extracting the teeth, I clipped off all the mucus tissue that filled the space between the necks of the teeth, leaving a smooth border. I then dissect back the fibrous mucus tissue from the alveolar wall or border, about one-fourth of an inch, and cut off with a pair of bone forceps the alveolar edge from a fourth to three-eighths of an inch deep; this will allow the edge of the mucus tissue to come together, having the appearance of a cut only around the dental arch. In the cases I have thus far prepared in this manner, I believe I saved the patients much inflammation and suffering, and also nature the task of removing this same matter by absorption or effusion. By this process we, in a measure, destroy the mucus excretory follicles around the dental border, and thereby they sooner become hard and firm. We shall save the risk of leaving detached pieces of bone in the gums, to find their way out by abscess or absorption. We shall save the patient most of the disagreeable effects from the effluence from the gums; we can save four months time in fitting the gums for a permanent set of teeth, and not least, in a pecuniary view of the subject, we have better shaped mouths, and consequently easily fitted and handsomer work when done. But the difference in age, health and number of teeth to be removed, should be the criterion for our government under this operation. I offer these remarks for what they may be worth, and hope to hear from some of our able dentists upon this, or any other mode of preparing the mouth for sets of teeth."

